# Unusual presentation of Lynch Syndrome

**DOI:** 10.1186/1897-4287-7-12

**Published:** 2009-06-03

**Authors:** Veronica PCC Yu, Marco Novelli, Stewart J Payne, Sam Fisher, Rebecca A Barnetson, Ian M Frayling, Ann Barrett, David Goudie, Audrey Ardern-Jones, Ros Eeles, Susan Shanley

**Affiliations:** 1 MRC Clinical Sciences Centre, Imperial College, Hammersmith Campus, Du Cane Road, London, W12 0NN, UK; 2 Department of Histopathology, University College London, Rockefeller Building, University Street, London, WC1E 6JJ, UK; 3 North West Thames Regional Genetics Service, Northwick Park & St Mark's Hospital, Watford Road, Harrow, London, HA1 3UJ, UK; 4 Colon Cancer Genetics Group, University of Edinburgh Cancer Research Centre and MRC Human Genetics Unit, Western General Hospital, Edinburgh, EH4 2XU, UK; 5 Institute of Medical Genetics, Cardiff University, School of Medicine, Heath Park, Cardiff, UK; 6 School of Medicine, Health Policy and Practice, University of East Anglia, Norwich, NR4 7TJ, UK; 7 Human Genetics, Department of Pathology, Ninewells Hospital and Medical School, Dundee, UK; 8 Cancer Genetics Unit, Royal Marsden NHS Foundation Trust, Fulham Road, London, SW3 6JJ, UK; 9 The Institute of Cancer Research, 15 Cotswold Rd, Sutton, Surrey, SM2 5NG, UK

## Abstract

Lynch Syndrome/HNPCC is a syndrome of cancer predisposition linked to inherited mutations of genes participating in post-replicative DNA mismatch repair (MMR). The spectrum of cancer associated with Lynch Syndrome includes tumours of the colorectum, endometrium, ovary, upper gastrointestinal tract and the urothelium although other cancers are rarely described. We describe a family of Lynch Syndrome with an *hMLH1 *mutation, that harbours an unusual tumour spectrum and its diagnostic and management challenges.

## Introduction

Lynch cancer family syndrome, also called hereditary non-polyposis colorectal cancer syndrome (HNPCC), is an autosomal dominant cancer predisposition disorder (OMIM: #14500). Lynch Syndrome carriers have an increased life-time risk of early onset colorectal cancer (24–80%) [1-3] and in women, endometrial cancer (20–60%) [1,3-5]. Other recognised cancer risks include: transitional cell carcinomata of the renal pelvis and ureters, carcinoma of the stomach, hepatobiliary and small intestinal cancers and ovarian carcinomas. Rarely other cancers have been described [6,7]. Sets of criteria (the revised Bethesda and the Amsterdam I and II criteria) for considering the diagnosis take into account the tumour spectrum in individuals and number of affected family members, age of tumour onset and pathological features of tumours.

Tumour predisposition in Lynch Syndrome is due to mutations in mismatch repair (MMR) genes. Though genes including *hMLH1*, *hMSH2*, *hMSH6*, *hPMS2*, *hMLH3*, *hMSH3 *and *hPMS1*, all participate in this process, approximately 90% of the germline mutations found in Lynch Syndrome families involve *hMLH1 *and *hMSH2 *[8,9]. More than 450 germline abnormalities of MMR genes have been described (InSiGHT, ). The majority of these mutations are recognized as pathogenic as they result in the expression of truncated proteins. However, at least 32% of *hMLH1 *mutations and 18% of *hMSH2 *mutations, are missense variants, which cause single amino-acid substitutions and are often of debatable relevance to pathogenicity [8].

Inactivation of the MMR system in Lynch Syndrome, due to somatic mutation of the corresponding normal allele, results in the accumulation of DNA replication errors in tumours associated with the syndrome. This is demonstrable as variation in the length of repetitive DNA sequences (microsatellites) in tumour-extracted DNA, a phenomenon known as microsatellite instability (MSI). Many Lynch syndrome-associated tumours also manifest loss of staining of the protein encoded by whichever MMR gene is mutated, making immunohistochemistry (IHC) of tumours (often together with microsatellite analysis) a helpful first step in the evaluation of a possible MMR-deficient tumour. This may be undertaken prior to determining if mutation searching of MMR genes in germline DNA is indicated.

The case presented here illustrates unusual phenotypes, which raise questions regarding optimal surveillance protocols in this and other Lynch syndrome families.

## Case report

**Figure 1:**
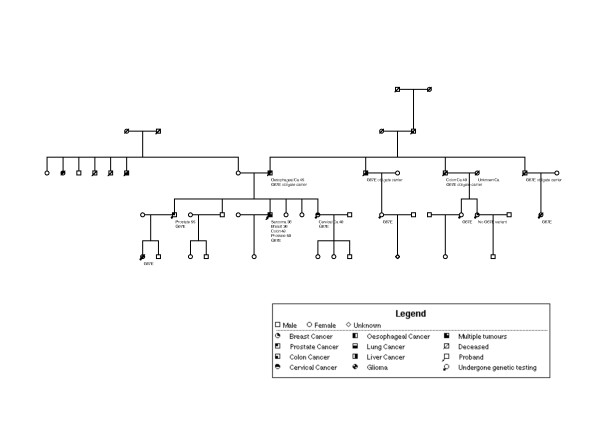
**Family tree**. The proband is indicated with an arrow. Nine mutation carriers are depicted as affected with cancer.

A 40-year old Northern European male was first seen in the genetics clinic in 1994. He had a history of leiomyosarcoma of the thigh at age 36, treated with a compartmental resection and post-operative radiotherapy and male breast cancer (stage T_2_N_0_M_0_) for which he had a right mastectomy. The relevant family history at presentation was a paternal history of a father (an ex-smoker but non-drinker) with oesophageal cancer at 47, and a paternal grandfather and two paternal uncles who had been affected with colon cancer in their forties (Figure 1).

Initial diagnoses considered included Li Fraumeni syndrome [9], which is associated with germline mutations in the *TP53 *gene, due to the presence of a sarcoma and breast cancer in the proband. Mutations in the *hMSH2 *gene, which had been recently cloned at that time were also considered as the paternal branch of the family including the uncles and grandfather fitted the Amsterdam I criteria. Full sequencing of the *TP53 *gene from blood DNA was uninformative and mutation testing for mismatch repair mutations was not available at that time. Subsequently, *BRCA2 *was considered a candidate gene because of the presence of male breast cancer, but no mutations were found in either *BRCA1 *or *BRCA2 *by screening of the full coding sequences of these genes with dHPLC and large rearrangement testing by MLPA.

**Figure 2:**
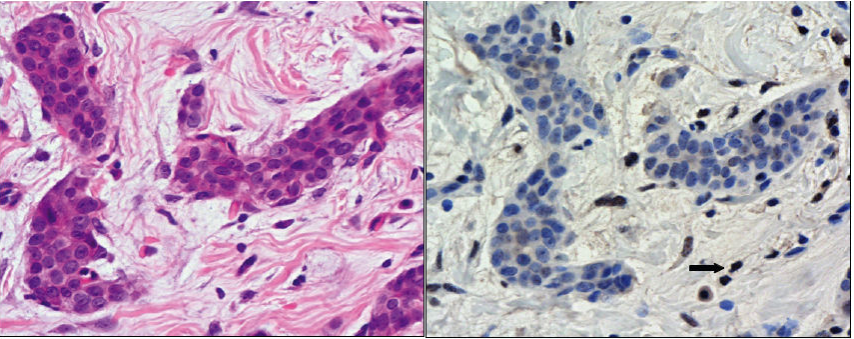
**The proband's leiomyosarcoma shows loss of MLH1 staining (right panel)**. Haematoxylin and Eosin of the section (left panel).

The patient went on to develop two further primaries – colon cancer at 48 years (a T_1 _tumour, treated with a sub-total colectomy) and prostate cancer at 50 years treated with brachytherapy. In addition, over the next decade the family history continued to evolve with the development of cervical adenosquamous carcinoma in his sister, an oligodendroglioma in his niece, a further case of colon cancer in a cousin and a prostate cancer in the proband's close relatives (Figure 1). Immunohistochemical studies of his breast carcinoma and sarcoma showed loss of hMLH1 staining (Figure 2) while hMSH2 and hMSH6 staining were normal. Staining was not available in the tumours from his immediate relatives, but an additional 8 cancers and one adenoma from other known mutation carriers in a different branch of the same family also showed loss of hMLH1 staining (data not shown) while hMSH2 and hMSH6 staining were normal. MSI testing was less extensively available in the family. While a high level of MSI was demonstrable in the proband's sarcoma (with three markers D5S346, D17S250, D2S123), it was not evident in his breast cancer, despite both tumours losing protein expression. This is likely to be a function of the known insensitivity of markers optimised for the detection of MSI in colorectal cancers, being used to try to detect MSI in other tumour types [10]. MSI was also demonstrated in 5 samples (4 cancers and one adenoma) from other known carriers in the second branch of the family.

Molecular analysis of the proband's germline DNA identified a missense variant in the *hMLH1 *gene (reference sequence NM_000249.2) in exon 2 at nucleotide position 200 (c.200G>A), which predicts substitution of glycine 67 with a glutamic acid residue (p.Gly67Glu). This putative p.Gly67Glu mutation segregates with cancer predisposition in nine family members (see Figure 1). The proband is currently well and has surveillance with annual sigmoidoscopy and upper gastrointestinal endoscopy, annual chest wall examination, 6 monthly and PSA level measurements and sarcoma follow-up.

## Discussion

This case illustrates some of the challenges in assessing and managing families where Lynch Syndrome is being considered. In particular, it illustrates the potential for tumours to arise that are not regarded as classic elements of the Lynch Syndrome-spectrum and the difficulty in determining optimal surveillance protocols for such families.

In this family, the identification of two unusual tumours (male breast cancer and sarcoma) concurrently at a young age raised the question of Li-Fraumeni syndrome [10] as these are classic tumours from that spectrum (although breast cancer in this syndrome is usually seen in females). Although the family do not fit the classic criteria for the syndrome, they do meet Li-Fraumeni like criteria [11].

The fact that the breast cancer occurred in a male proband who subsequently developed prostate cancer also raised the possibility of his having an unusual presentation of a *BRCA2 *mutation, as lifetime risk (to age 70) of these tumours is increased to about 7% and 20% respectively [12-15]. The strength of the family history of colorectal cancer would not, however, be in keeping with the slight increases in colorectal risk seen with BRCA mutations; these are usually seen in those with *BRCA1 *mutations rather than *BRCA2 *[16,17].

The family does, however, fit the revised Amsterdam criteria, which suggests a diagnosis of Lynch Syndrome. The evidence for pathogenicity of the missense *hMLH1 *variant is extensive. It includes the loss of hMLH1 protein staining in our proband's tumours and in the additional nine tumours (8 cancers and one adenoma) from his distant relatives. This loss of hMLH1 staining may be due more to a loss of antibody-specific epitope rather than an actual absence of protein expression. The co-segregation of the variant with disease similarly supports pathogenicity as does recent *in vivo *demonstration of microsatellite instability in a yeast model of this variant [18]. The p.Gly67Glu mutation is not identified in 1688 unaffected controls [19]. In addition, *in-silico *analysis using three phenotype predictive tools – Align GVGD , PolyPhen  and SIFT , all support p.Gly67Glu being pathogenic.

There have been three reported cases of p.Gly67Arg mutations [19,20] which are regarded as pathogenic because the Gly67 residue is located in the catalytic ATP-binding pocket of MLH1. This is evolutionarily highly conserved and essential for its biological function [20]. Tumours from p.Gly67Arg are often associated with MSI, and this mutation segregates with disease.

Our proband has been previously mentioned in a report of Lynch Syndrome variants [18] but we are not aware of other descriptions of the co-existence of breast tumour, sarcoma, colorectal cancer and prostate cancer in a single proband with an *hMLH1 *mutation. Such tumours have, however, been reported in isolation in Lynch Syndrome patients. Soft tissue sarcomas have been described in Lynch Syndrome families [21,22] and may be associated with mutations in *hMSH2, hMSH6 and MLH1 *[22,23]. Male breast cancer has been reported in an *hMLH1 *carrier family [7] and the breast cancer risk for women in *hMLH1 *families has been reported as being increased by Scott *et al*. [24] although others have not replicated this [25,26]. Recently, data from Barrow *et al*. [26] support a possible moderate increase in these families. Similarly, prostate cancers have also been reported in Lynch Syndrome kindreds [27,28] but as we do not have staining information for the prostate tumours (nor for the cervical cancer) in our family we cannot know if mismatch repair was involved in their pathogenesis.

Possible explanations for the range of tumours seen in our patient could include unidentified modifier genes [25] that have increased his risks, but have not been co-inherited by other *hMLH1 *mutation carriers in his family. Alternatively, it remains possible that there is an effect specific to mutations at this location as it is noted that the closely related p.Gly67Trp mutation has also been reported in a carrier to present with colorectal cancer, brain cancer, lymphoma, leukemia and rhabdomyosarcoma [29].

We cannot exclude that the patient's later cancers were radiation induced; there is scatter dose and so second tumours can occur both within and outside the radiation field. Of note, homozygous *Mlh1*-deficient mice have increased radiation sensitivity [30] and increased chromatid exchanges have been induced in G2 in lymphocytes from Lynch Syndrome patients [31].

The glioma in the proband's niece, although rare, is a recognised element of Lynch Syndrome families, who may be referred to as having the Turcot variant.

Due to the rarity of such clinical presentations, there are few data on which to base advice regarding modifications to the screening regime for *hMLH1 *carriers in this family. In addition to surveillance of his gastrointestinal tract (with annual sigmoidoscopy and gastroscopy (due to his paternal history of oesophageal cancer), our patient has 6 monthly follow up of his multiple primaries with a general physical examination including that of his chest wall, and PSA levels. He has urinary cytology performed as follow up of an episode of atypia that was attributed to his prostate therapy but the value of such cytology is debatable given the lack of sensitivity demonstrated by Myrhoj *et al*. [32] and may be discontinued.

We have suggested that unaffected carriers are offered two yearly colonoscopy from 25 and second yearly gastroscopy from 35 (due to the diagnosis of oesophageal cancer in the proband's father at age 47) and that women have gynaecological surveillance with endometrial biopsy from 35 and ultrasound of the ovaries with CA125 as part of the research programme UKFOCSS (United Kingdom Familial Ovarian Cancer Screening Study). The screening modifications based on the cancer history specifically in the proband have included annual PSA screening from 45 and the advice that male carriers report any changes in breast tissue early, although it is difficult to know if such advice is pertinent more widely than within our proband's immediate relatives.

In summary, we describe a family kindred where an *hMLH1 *p.Gly67Glu germline mutation is associated with an unusual presentation of Lynch Syndrome. Identification of more families with this pathogenic mutation is likely to shed light on the mechanism for carcinogenesis in our kindred.

## Consent

Written consent has been obtained by the family involved.

Family tree shown in figure 1 has been altered to maintain confidentiality without affecting interpretation of data.

## Competing interests

The authors declare that they have no competing interests.

## References

[B1] Aarnio M, Mecklin JP, Aaltonen LA, Nystrom-Lahti M, Jarvinen HJ (1995). Life-time risk of different cancers in hereditary non-polyposis colorectal cancer (HNPCC) syndrome. Int J Cancer.

[B2] Lynch HT, Smyrk T (1996). Hereditary nonpolyposis colorectal cancer (Lynch syndrome). An updated review. Cancer.

[B3] Vasen HF, Moslein G, Alonso A, Bernstein I, Bertario L, Blanco I, Burn J, Capella G, Engel C, Frayling I (2007). Guidelines for the clinical management of Lynch syndrome (hereditary non-polyposis cancer). J Med Genet.

[B4] Aarnio M, Sankila R, Pukkala E, Salovaara R, Aaltonen LA, de laChapelle A, Peltomaki P, Mecklin JP, Jarvinen HJ (1999). Cancer risk in mutation carriers of DNA-mismatch-repair genes. Int J Cancer.

[B5] Watson P, Vasen HF, Mecklin JP, Jarvinen H, Lynch HT (1994). The risk of endometrial cancer in hereditary nonpolyposis colorectal cancer. Am J Med.

[B6] Lynch HT, Deters CA, Hogg D, Lynch JF, Kinarsky Y, Gatalica Z (2003). Familial sarcoma: challenging pedigrees. Cancer.

[B7] Boyd J, Rhei E, Federici MG, Borgen PI, Watson P, Franklin B, Karr B, Lynch J, Lemon SJ, Lynch HT (1999). Male breast cancer in the hereditary nonpolyposis colorectal cancer syndrome. Breast Cancer Res Treat.

[B8] Peltomaki P, Vasen H (2004). Mutations associated with HNPCC predisposition – Update of ICG-HNPCC/INSiGHT mutation database. Dis Markers.

[B9] Lynch HT, de la Chapelle A (1999). Genetic susceptibility to non-polyposis colorectal cancer. J Med Genet.

[B10] Li FP, Fraumeni JF (1969). Soft-tissue sarcomas, breast cancer, and other neoplasms. A familial syndrome?. Ann Intern Med.

[B11] Birch JM, Hartley AL, Tricker KJ, Prosser J, Condie A, Kelsey AM, Harris M, Jones PH, Binchy A, Crowther D (1994). Prevalence and diversity of constitutional mutations in the p53 gene among 21 Li-Fraumeni families. Cancer Res.

[B12] Ford D, Easton DF, Stratton M, Narod S, Goldgar D, Devilee P, Bishop DT, Weber B, Lenoir G, Chang-Claude J (1998). Genetic heterogeneity and penetrance analysis of the BRCA1 and BRCA2 genes in breast cancer families. The Breast Cancer Linkage Consortium. Am J Hum Genet.

[B13] The Breast Cancer Linkage Consortium (1999). Cancer risks in BRCA2 mutation carriers. J Natl Cancer Inst.

[B14] Thompson D, Easton D (2001). Variation in cancer risks, by mutation position, in BRCA2 mutation carriers. Am J Hum Genet.

[B15] Tai YC, Domchek S, Parmigiani G, Chen S (2007). Breast cancer risk among male BRCA1 and BRCA2 mutation carriers. J Natl Cancer Inst.

[B16] Niell BL, Rennert G, Bonner JD, Almog R, Tomsho LP, Gruber SB (2004). BRCA1 and BRCA2 founder mutations and the risk of colorectal cancer. J Natl Cancer Inst.

[B17] Thompson D, Easton DF (2002). Cancer Incidence in BRCA1 mutation carriers. J Natl Cancer Inst.

[B18] Clyne M, Offman J, Shanley S, Virgo JD, Radulovic M, Wang Y, Ardern-Jones A, Eeles R, Hoffmann E, Yu VP (2009). The G67E mutation in hMLH1 is associated with an unusual presentation of Lynch syndrome. Br J Cancer.

[B19] Barnetson RA, Cartwright N, van Vliet A, Haq N, Drew K, Farrington S, Williams N, Warner J, Campbell H, Porteous ME (2008). Classification of ambiguous mutations in DNA mismatch repair genes identified in a population-based study of colorectal cancer. Hum Mutat.

[B20] Shimodaira H, Filosi N, Shibata H, Suzuki T, Radice P, Kanamaru R, Friend SH, Kolodner RD, Ishioka C (1998). Functional analysis of human MLH1 mutations in Saccharomyces cerevisiae. Nat Genet.

[B21] Medina Arana V, Barrios del Pino Y, Garcia-Castro C, Gonzalez-Aguilera JJ, Fernandez-Peralta A, Gonzalez Hermoso F (2002). Highly aggressive leiomyosarcoma associated with Lynch II syndrome: increasing the range of extracolonic cancers related with hereditary non-polyposis colonic cancer. Ann Oncol.

[B22] Nilbert M, Therkildsen C, Nissen A, Akerman M, Bernstein I (2009). Sarcomas associated with hereditary nonpolyposis colorectal cancer: broad anatomical and morphological spectrum. Fam Cancer.

[B23] Hirata K, Kanemitsu S, Nakayama Y, Nagata N, Itoh H, Ohnishi H, Ishikawa H, Furukawa Y (2006). A novel germline mutation of MSH2 in a hereditary nonpolyposis colorectal cancer patient with liposarcoma. Am J Gastroenterol.

[B24] Scott RJ, McPhillips M, Meldrum CJ, Fitzgerald PE, Adams K, Spigelman AD, du Sart D, Tucker K, Kirk J (2001). Hereditary nonpolyposis colorectal cancer in 95 families: differences and similarities between mutation-positive and mutation-negative kindreds. Am J Hum Genet.

[B25] Scott RJ (2008). Modifier Genes and HNPCC: Variable phenotypic expression in HNPCC and the search for modifier genes. Eur J Hum Genet.

[B26] Barrow E, Robinson L, Alduaij W, Shenton A, Clancy T, Lalloo F, Hill J, Evans DG (2009). Cumulative lifetime incidence of extracolonic cancers in Lynch syndrome: a report of 121 families with proven mutations. Clin Genet.

[B27] Fredriksson H, Ikonen T, Autio V, Matikainen MP, Helin HJ, Tammela TL, Koivisto PA, Schleutker J (2006). Identification of germline MLH1 alterations in familial prostate cancer. Eur J Cancer.

[B28] Soravia C, Klift H van der, Brundler MA, Blouin JL, Wijnen J, Hutter P, Fodde R, Delozier-Blanchet C (2003). Prostate cancer is part of the hereditary non-polyposis colorectal cancer (HNPCC) tumor spectrum. Am J Med Genet.

[B29] Wang Q, Lasset C, Desseigne F, Saurin JC, Maugard C, Navarro C, Ruano E, Descos L, Trillet-Lenoir V, Bosset JF, Puisieux A (1999). Prevalence of germline mutations of hMLH1, hMSH2, hPMS1, hPMS2, and hMSH6 genes in 75 French kindreds with nonpolyposis colorectal cancer. Hum Genet.

[B30] Tokairin Y, Kakinuma S, Arai M, Nishimura M, Okamoto M, Ito E, Akashi M, Miki Y, Kawano T, Iwai T, Shimada Y (2006). Accelerated growth of intestinal tumours after radiation exposure in Mlh1-knockout mice: evaluation of the late effect of radiation on a mouse model of HNPCC. Int J Exp Pathol.

[B31] Franchitto A, Pichierri P, Genuardi M, De Santis A, Palitti F (2001). Investigation of G2-phase chromosomal radiosensitivity in hereditary non-polyposis colorectal cancer cells. Int J Radiat Biol.

[B32] Myrhoj T, Andersen MB, Bernstein I (2008). Screening for urinary tract cancer with urine cytology in Lynch syndrome and familial colorectal cancer. Fam Cancer.

